# Development of a convolutional neural network for the identification and the measurement of the median nerve on ultrasound images acquired at carpal tunnel level

**DOI:** 10.1186/s13075-022-02729-6

**Published:** 2022-02-08

**Authors:** Gianluca Smerilli, Edoardo Cipolletta, Gianmarco Sartini, Erica Moscioni, Mariachiara Di Cosmo, Maria Chiara Fiorentino, Sara Moccia, Emanuele Frontoni, Walter Grassi, Emilio Filippucci

**Affiliations:** 1 Rheumatology Unit, Department of Clinical and Molecular Sciences, Polytechnic University of Marche, “Carlo Urbani” Hospital, Via Aldo Moro 25, 60035 Jesi, Ancona Italy; 2 Department of Information Engineering, Polytechnic University of Marche, Ancona, Italy; 3 The BioRobotics Institute and Department of Excellence in Robotics and AI, Scuola Superiore Sant’Anna, Pisa, Italy

## Abstract

**Background:**

Deep learning applied to ultrasound (US) can provide a feedback to the sonographer about the correct identification of scanned tissues and allows for faster and standardized measurements. The most frequently adopted parameter for US diagnosis of carpal tunnel syndrome is the increasing of the cross-sectional area (CSA) of the median nerve. Our aim was to develop a deep learning algorithm, relying on convolutional neural networks (CNNs), for the localization and segmentation of the median nerve and the automatic measurement of its CSA on US images acquired at the proximal inlet of the carpal tunnel.

**Methods:**

Consecutive patients with rheumatic and musculoskeletal disorders were recruited. Transverse US images were acquired at the carpal tunnel inlet, and the CSA was manually measured. Anatomical variants were registered. The dataset consisted of 246 images (157 for training, 40 for validation, and 49 for testing) from 103 patients each associated with manual annotations of the nerve boundary. A Mask R-CNN, state-of-the-art CNN for image semantic segmentation, was trained on this dataset to accurately localize and segment the median nerve section. To evaluate the performances on the testing set, precision (*Prec*), recall (*Rec*), mean average precision (*mAP*), and Dice similarity coefficient (*DSC*) were computed. A sub-analysis excluding anatomical variants was performed. The CSA was automatically measured by the algorithm.

**Results:**

The algorithm correctly identified the median nerve in 41/49 images (83.7%) and in 41/43 images (95.3%) excluding anatomical variants. The following metrics were obtained (with and without anatomical variants, respectively): Prec 0.86 ± 0.33 and 0.96 ± 0.18, Rec 0.88 ± 0.33 and 0.98 ± 0.15, mAP 0.88 ± 0.33 and 0.98 ± 0.15, and DSC 0.86 ± 0.19 and 0.88 ± 0.19. The agreement between the algorithm and the sonographer CSA measurements was excellent [ICC 0.97 (0.94–0.98)].

**Conclusions:**

The developed algorithm has shown excellent performances, especially if excluding anatomical variants. Future research should aim at expanding the US image dataset including a wider spectrum of normal anatomy and pathology. This deep learning approach has shown very high potentiality for a fully automatic support for US assessment of carpal tunnel syndrome.

## Background

Carpal tunnel syndrome (CTS) is commonly encountered in rheumatology daily practice, and it is a frequent condition also in other healthcare settings such as orthopedics, neurology, physiatry, and primary care [1]. It is defined as the constellation of signs and symptoms due to the compression of the median nerve while it passes through the carpal tunnel [2]. Even if the diagnosis relies on clinical history and physical examination, confirmatory tests such as nerve conduction studies and ultrasound (US) are often used [2].

In particular, US is helpful in assisting the diagnosis of CTS [3–5], and it adds value to electrodiagnosis, being capable of identifying pathological median nerve swelling as well as the cause of the compression of the median nerve (e.g., flexor tendons tenosynovitis, wrist synovitis, tophi, or persistent median artery thrombosis) [6–9].

The most frequently adopted parameter for US diagnosis of CTS is the increasing of the cross-sectional area (CSA) of the median nerve measured at the proximal inlet of the carpal tunnel (at the level of the pisiform bone) [10, 11].

Some of the limitations to the spread of US in rheumatology are its operator dependency, the need for a supervised training, and the inter-observer variability in obtaining standardized measurements [12].

Recently, artificial intelligence (AI), and in particular deep learning (DL), applied to US has demonstrated to be a possible solution to some of these issues. In fact, it can provide an immediate feedback to the beginner sonographer about the correct identification of scanned tissues and allows for faster and more standardized measurements [13–17].

In spite of such promising results, only a relatively small number of studies have applied DL to US in the field of rheumatic and musculoskeletal diseases [18–22], and the contributions focusing on its application on the US assessment of CTS are even fewer [23, 24].

DL is a class of AI algorithms that is inspired by the structure of the human brain, capable of autonomous learning and composed by many layers of artificial neurons that extract higher-level features from data [25]. Convolutional neural networks (CNNs) are DL algorithms designed for processing structured arrays of data such as images, suited to solve various image analysis tasks, such as object classification, detection, and segmentation, in a variety of different fields, including medical image analysis [25, 26].

The main aim of the present study was to develop an end-to-end CNN, i.e., Mask R-CNN [27], for the localization and segmentation of the median nerve and the automatic measurement of its CSA on US images acquired at the inlet of the carpal tunnel.

## Materials and methods

### US image acquisition, interpretation, and annotation

Consecutive patients with rheumatic and musculoskeletal disorders were recruited at the Rheumatology Unit of “Carlo Urbani” Hospital in Jesi, Italy. Patients < 18 years old were excluded. The study was conducted in accordance with the Helsinki Declaration and was approved by the local ethics committee (Comitato Etico Regione Marche, number 262). All patients signed informed consent.

Basic clinical and demographic data were collected. The US assessment was performed by one of three sonographers with different degrees of experience in musculoskeletal US (G.Sa.: 1 month with a dedicated intensive training; G.Sm.: 4 years; E.Fi.: more than 20 years of experience) with a MyLab Class C (Esaote Spa, Genoa, Italy) US system equipped with a linear 6–18-MHz probe.

Patients were seated in a comfortable position, with the forearm resting supine on the examination bed and fingers in neutral position. Each wrist was scanned in transverse views according with the 2017 EULAR standardized procedures for US imaging in rheumatology [28]. Representative images were acquired bilaterally at the carpal tunnel proximal inlet (at the level of the pisiform bone). The sonographer measured the CSA by tracing a continuous line within the hyperechogenic boundary of the nerve (along the internal margin of the epineurium). The presence of the following anatomical variants was registered: bifid median nerve, persistent median artery, and accessory muscles within the carpal tunnel. In the case of the bifid median nerve, the CSA was measured summing the areas of both branches.

All the images were reviewed by the expert sonographer (E.Fi.), and images not considered informative due to insufficient quality were excluded. The boundary of the nerve was manually annotated in each image of the dataset by the same operator (G.Sa.)

### CNN algorithm training and training strategy

In this work, Mask R-CNN [27] was trained for median nerve semantic segmentation from US images. Mask R-CNN integrates object detection task, where the goal is to detect object class along with bounding box prediction in an image, and consequently semantic segmentation task, which classifies each pixel into pre-defined categories. Thus, it enables to segment precisely median nerve boundaries, once learned its location in the US image.

**Fig. 1:**
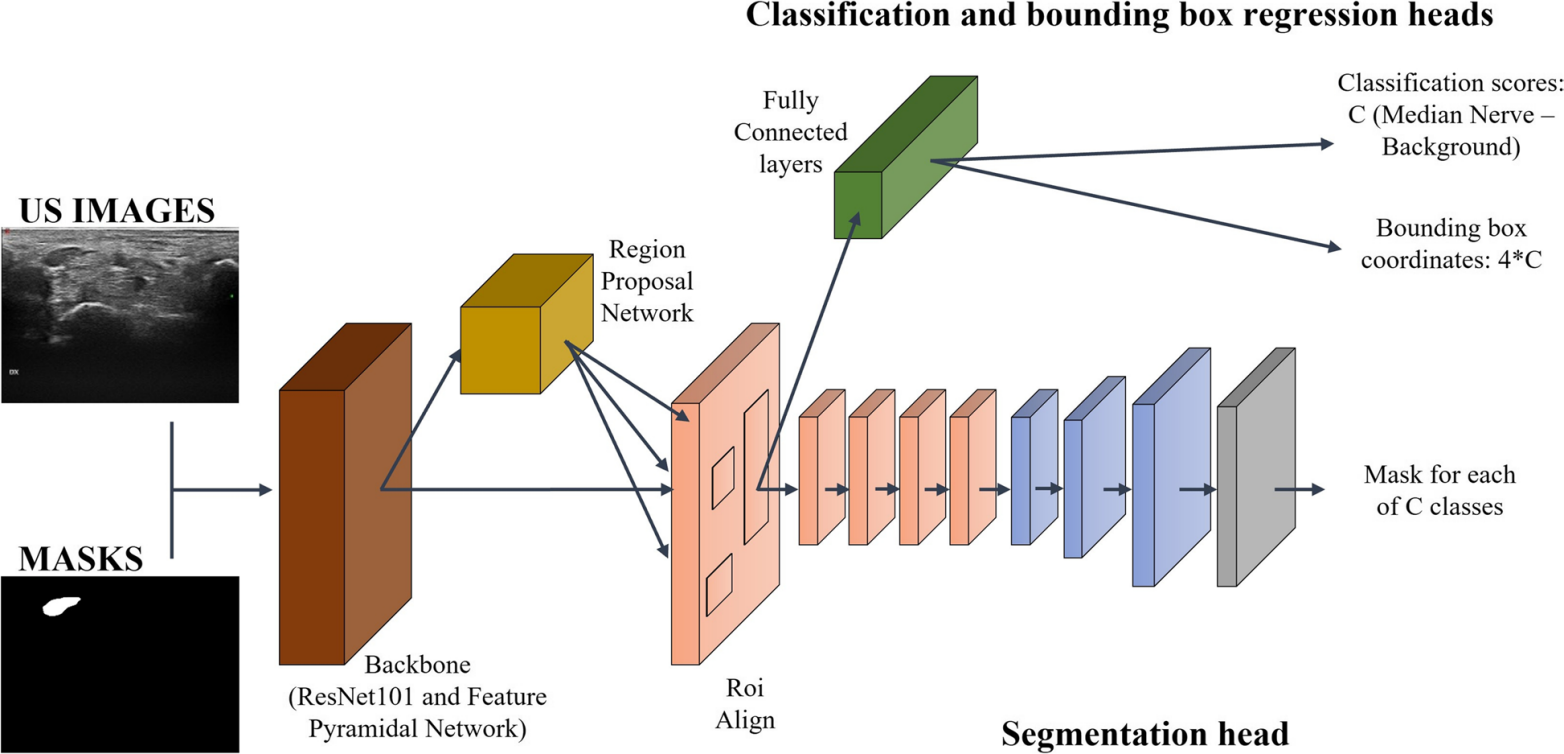
Mask R-CNN is a CNN made of backbone (composed by a ResNet101 and a feature pyramid network (FPN)), a region proposal network (RPN), ROIAlign, and three heads, for classification, bounding-box regression, and segmentation

This DL algorithm mainly works in two stages: first, it generates proposals about the regions where the target object might be based on the input image; second, it predicts the object class and its location refining the bounding box and from that its contours generating a mask in pixel level of the object based on the first stage proposal. A schematic representation of this end-to-end deep learning algorithm is shown in Fig. 1.

More specifically, we implemented a ResNet101 [29] combined with the feature pyramid network (FPN) [30], as backbone, to extract features from the input image at multiple levels. Subsequently, the region proposal network (RPN) generates proposals, then selected and processed by the ROIAlign layer that resizes them to a constant output dimension before passing them to the Mask R-CNN heads [27]. From the original Mask R-CNN implementation, we increased the output resolution of the segmentation head using three transposed convolutions with 256 2 × 2 filters and activated with the rectified linear unit (ReLU), instead of only one, to cope with the fragmented and low-contrasted edges of the median nerve.

The dataset consisted of 246 images from 103 patients, associated with manual annotations, which were used as ground truth to teach the algorithm to correctly localize and segment the median nerve. For this purpose, the dataset was split over patients in three parts: 157 images from 64 subjects for training, 40 images from 16 subjects for validation, and 49 images from 23 subjects for testing.

From the original size of 606 × 468 pixels, each US image and the corresponding annotation mask were resized to 512 × 512 pixels and zero-padded at right-most and bottom-most edges to get squared images with a size multiple of 32, as required by the FPN, while preserving the original aspect ratio.

To make up for the relatively small size of our dataset, weights computed on the COCO dataset [31] were used to initialize all layers of the model except for the input layers of the network heads and during training on-the-fly data augmentation was performed by randomly scaling in the range of (0.8, 1.2) and random translating in the range (− 0.2, 0.2) in both directions and performing random rotation between − 10 and 10 and shearing between − 2 and 2.

The training was performed using the Stochastic Gradient Descent as an optimizer for 150 epochs with an initial learning rate of 0.001 and momentum of 0.9.

A total of 256 anchors per image was used, with varying sizes (32, 64, 128, 256, and 512) and aspect ratios (1:1, 2:1, 1:2). These values were chosen considering the median nerve section dimension. The ROIAlign resized proposals to a fixed size of 14 × 14. Hence, the output of the proposed segmentation had a resolution of 112 × 112.

The model was trained using a multi-task cross-entropy loss function combining the loss of classification, localization, and segmentation mask: *L* = *L*_cls_ + *L*_bbox_ + *L*_mask_, where *L*_cls_, *L*_bbox_, and *L*_mask_ are respectively class, bounding box, and mask losses [27].

### Performance metrics

We considered a true positive (*TP*) prediction if the predicted bounding box overlapped the ground truth for at least 70% and had confidence higher than 0.98. Otherwise, the nerve detection was considered as false positive (*FP*). We considered a false negative (*FN*) when no bounding box was predicted at all.

To evaluate the performance in median nerve localization, precision (*Prec*) and recall (*Rec*) were computed as follows:

$${\displaystyle \begin{array}{l}\mathrm{Prec}=\frac{\mathrm{TP}}{\mathrm{TP}+\mathrm{FP}}\\ {}\mathrm{Rec}=\frac{\mathrm{TP}}{\mathrm{TP}+\mathrm{FP}}\end{array}}$$

The mean average precision (*mAP*), which represents the average of the area under the recall-precision curve, was also computed.

The median nerve segmentation performance was measured using the Dice similarity coefficient (*DSC*), which is defined as follows:

$$\mathrm{DSC}=\frac{2\times \left|{A}_{\mathrm{gt}}\cap {A}_{\mathrm{mask}}\right|}{\left|{A}_{\mathrm{gt}}\ \right|+\left|{A}_{\mathrm{mask}}\right|}$$

where *A*_gt_ and *A*_mask_ are the ground truth and predicted segmentation, respectively. When computing the *DSC*, only *TP*s were considered.

*Prec*, *Rec*, *mAP*, and *DSC* values can range between a minimum of 0 and a maximum of 1.

We also calculated the percentage of the images of the testing set in which the algorithm correctly identified only the true median nerve (*TP* prediction in the absence of a concomitant *FN* prediction in the same image).

### CSA automatic measurement

The CSA was automatically calculated from the median nerve section predicted by the algorithm, knowing the dimensions of a single pixel (0.062 mm × 0.062 mm) composing the US images. The CSA was calculated only on TP predictions, excluding images with FP predictions.

### Statistical analysis

The results are expressed as number and/or corresponding percentage for qualitative variables and as mean and standard deviation (SD) for quantitative variables. The chi-square test and the Mann-Whitney test were used to compare the qualitative and quantitative variables, respectively. The agreement in the CSA measurements between the operator (i.e., the gold standard) and the algorithm was calculated using a two-way mixed-effects intraclass correlation coefficient (ICC) with 95% confidence interval (CI).

The ICC is regarded as excellent if above 0.9, as good if between 0.75 and 0.9, as fair if between 0.4 and 0.75, and as poor if below 0.4.

## Results

### Patient characteristics

A total of 103 rheumatic patients were consecutively included in this single-center and cross-sectional study. Table 1 shows the main demographic characteristics of the participants.

**Table 1:** Demographic and clinical characteristics of 103 patients with rheumatic and musculoskeletal disorders included

Variable	Value
Age (years), mean ± SD	56 ± 13
Male/female ratio	1:1.8
BMI (kg/m^2^), mean ± SD	26.1 ± 4.5
Disease, *n* (%)	
Rheumatoid arthritis	23 (22%)
Osteoarthritis	19 (18%)
Psoriatic arthritis	18 (17%)
Fibromyalgia	11 (11%)
Systemic sclerosis	6 (6%)
Systemic lupus erythematosus	5 (5%)
CPPD	4 (4%)
Sjogren’s syndrome	3 (3%)
Polymyalgia rheumatica	3 (3%)
Others	11 (11%)

*Abbreviations*: *BMI* body mass index, *CPPD* calcium pyrophosphate deposition disease, *SD* standard deviation

Twenty-two out of 103 patients (21%) had a clinical diagnosis of CTS (10 unilateral, 13 bilateral).

### Performance metrics in the identification of the median nerve

The algorithm made 43 correct predictions (*TP*), two *FN* predictions, and six *FP* predictions.

Table 2 shows the results of the performance metrics for the localization (*Prec*, *Rec*, and *mAP*) and the segmentation (*DSC*) of the median nerve.

**Table 2:** Performance metrics of the convolutional neural network (CNN) algorithm for the localization and segmentation of the median nerve

	Prec	Rec	mAP	DSC
**49 testing images**	0.86 ± 0.33	0.88 ± 0.33	0.88 ± 0.33	0.86 ± 0.19

Results are expressed as mean ± standard deviation

*DSC* Dice similarity coefficient, *mAP* mean average precision, *Prec* precision, *Rec* recall

**Fig. 2:**
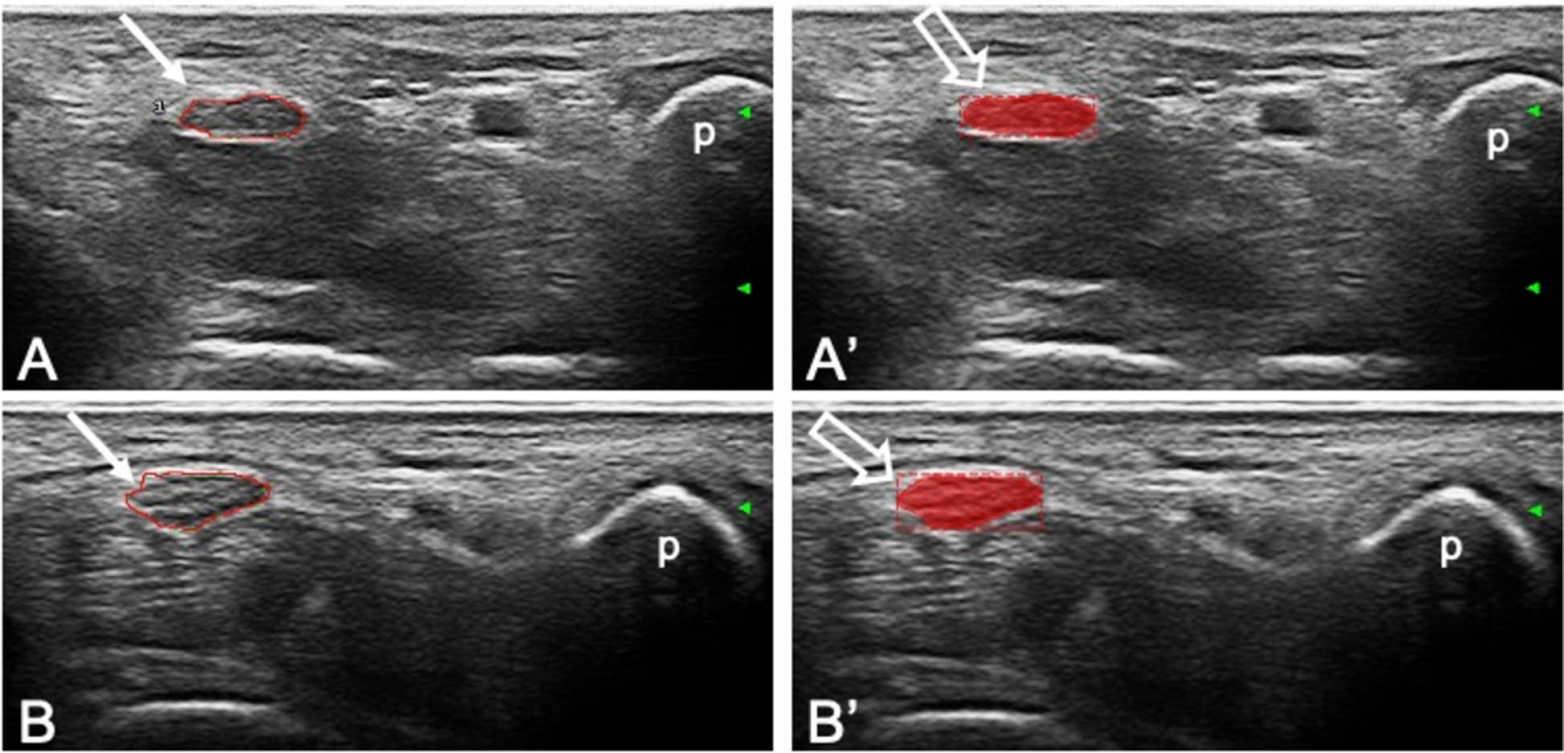
Correct localization and segmentation of the median nerve. Transverse scans acquired at the carpal tunnel proximal inlet in two patients (A-A′ and B-B′) showing in the left panels (A and B) the manual annotations of the boundary of the median nerve made by the operator (arrows) and in the right panels (A′ and B′) the corresponding predictions made by the algorithm (open arrows). p, pisiform bone

Overall, the algorithm correctly identified and segmented the median nerve in 41 out of 49 images (83.7%) (Fig. 2).

**Fig. 3:**
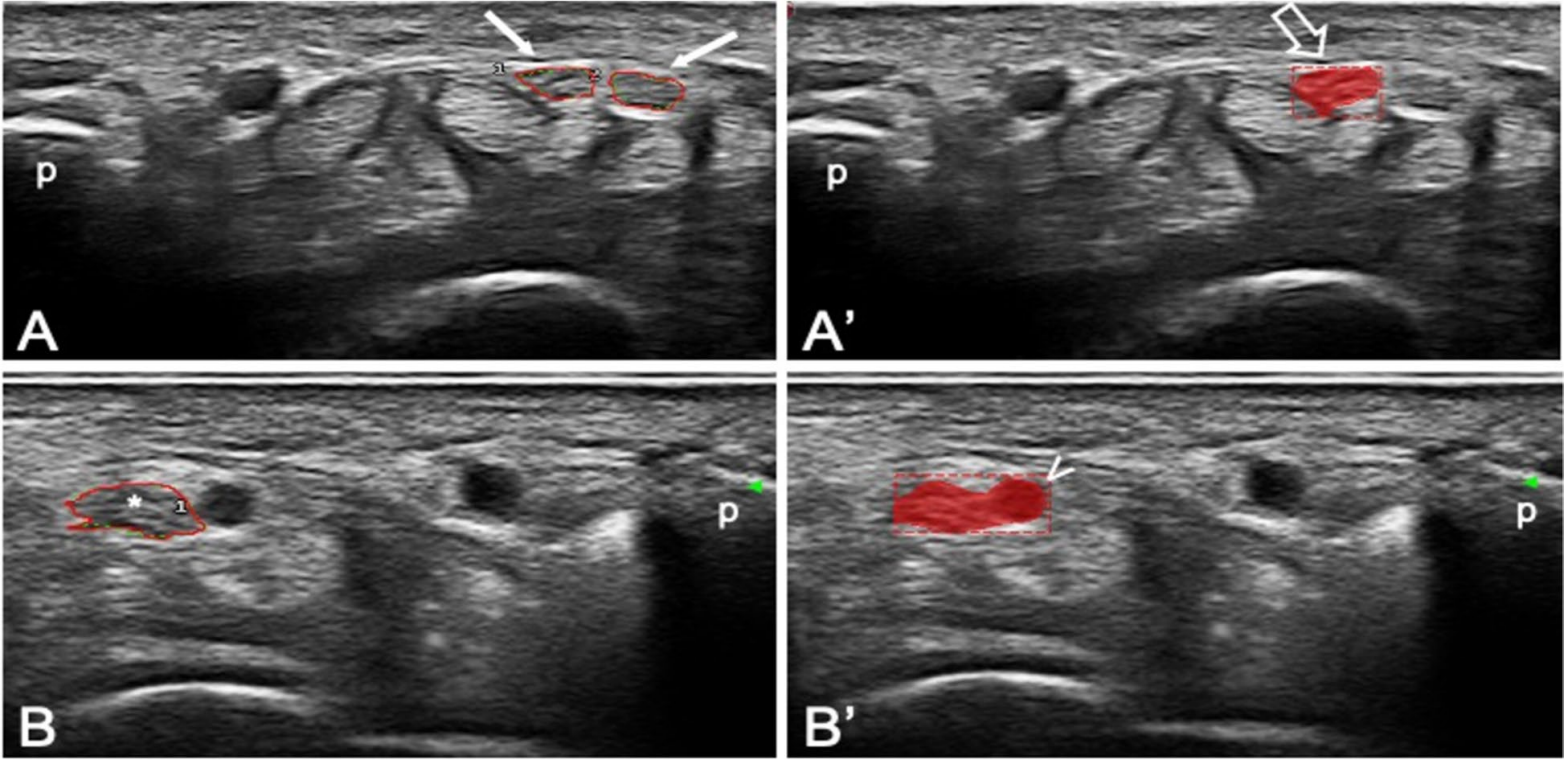
Representative images of incorrect predictions. Transverse scans acquired at the carpal tunnel proximal inlet in two patients showing the correct identification of only one branch (open arrow) of a bifid median nerve (arrows) (A-A′) and the wrong inclusion of an adjacent vessel (arrowhead) in the prediction of the median nerve (asterisk) (B-B′). p, pisiform bone

After a revision by the expert sonographers of the cause of the errors made by the algorithm, it was noted that the algorithm underperformed when it was asked to interpret images with anatomical variants (i.e., bifid median nerve or prominent persistent median artery). A sample of US images of the testing set containing anatomical variants is represented in Fig. 3.

Thus, we performed a sub-analysis of the performance metrics excluding such relatively rare anatomical variants from the testing set (*n* = 6). These results are shown in Table 3. In such sub-analysis, the algorithm correctly identified and segmented the median nerve in 41 out of 43 images (95.3%). In both images with incorrect predictions, the algorithm considered a nearby tendinous structure as another branch of the median nerve (i.e., the flexor carpi radialis tendon in one image and one of the finger flexor tendons in the other). These incorrect predictions may be related to the fact that in the transverse US view, the tendons show an oval shape, which is very similar to the one of the median nerve.

**Table 3:** Performance metrics of the convolutional neural network (CNN) algorithm for the localization and segmentation of the median nerve in images without anatomical variants (i.e., bifid median nerve or prominent persistent median artery)

	Prec	Rec	mAP	DSC
**43 testing images**	0.96 ± 0.18	0.98 ± 0.15	0.98 ± 0.15	0.88 ± 0.19

Results are expressed as mean ± standard deviation

*DSC* Dice similarity coefficient, *mAP* mean average precision, *Prec* precision, *Rec* recall

We trained and tested the algorithm using TensorFlow on a GPU GeForce RTX 2080, and the average inference time was 1.7 s, which could be further reduced with computational resources higher than the ones available for our study.

### CSA automatic measurement

The average CSA measured by the operator was 10.4 ± 4.6 mm^2^ while the average CSA automatically calculated by the algorithm was 10.4 ± 4.3 mm^2^, with no significant difference (*p* = 0.88).

The agreement between the automatic algorithm measurement and the sonographer manual measurement of the CSA was excellent [ICC 0.97 (95% CI 0.94–0.98)].

## Discussion

In the last decades, US has largely demonstrated its usefulness in several aspects of the management of patients with rheumatic diseases [32–34]. It is safe, cost-effective, readily accessible, and generally well tolerated by the patients. Despite all the above, US is still far from being systematically adopted in rheumatology daily practice. This is largely due to its operator dependency and the intrinsic difficulties in standardizing measurements which may undermine its reproducibility.

DL has the potential to fill these gaps, facilitating a self-teaching approach not requiring continuous supervision by an expert sonographer and increasing standardization and reproducibility.

DL application to US is of particular interest in the setting of CTS assessment. In fact, CTS is a frequent condition encompassing many different medical specialties such as rheumatology, orthopedics, neurology, physiatry, and primary care. There is a growing body of evidence highlighting the value of US detection of the thickening of the median nerve (measured with the CSA obtained at the proximal inlet of carpal tunnel) as a confirmatory diagnostic test on top of clinical examination. Moreover, in patients with CTS, differently from electrophysiology, US can demonstrate the cause of the compression of the median nerve (e.g., tenosynovitis) [7].

However, despite this, US is struggling to find its place in the diagnostic work-up, being rarely preferred to or carried out together with electrodiagnostic tests. This may be partially due to the lack of the competencies needed to perform and interpret an US examination at the carpal tunnel level among different specialists facing CTS.

The correct identification of the median nerve is the first step for any beginner sonographer that approaches CTS assessment. The distinction of the median nerve from the finger flexor tendons is not an easy task due to the similar round shape on the transverse view. Sonographic criteria for the identification of peripheral nerve morphology were proposed by Silvestri et al. [35]. The authors stated that on transverse scans, peripheral nerves, including the median nerve, are characterized by the presence of multiple rounded hypoechoic areas with a homogeneous hyperechoic background (i.e., fascicular pattern). The fascicular pattern characteristic of nerves may be differentiated from tendons’ fibrillar pattern (linear hypo- and hyperechoic areas on transverse scan).

Such pattern recognition is a well-suited task for DL. Thus, we proposed an end-to-end DL approach to median nerve segmentation from US images acquired into daily clinical practice.

Our results show that the developed CNN algorithm is accurate in the identification and segmentation of median nerve on transverse US images acquired at the proximal inlet of the carpal tunnel.

If implemented on US machines, it would offer an almost real-time automatic feedback to the beginner sonographer approaching CTS US, avoiding the need of continuous supervision by an expert sonographer.

Furthermore, the present CNN algorithm is not only useful for the unexperienced sonographer. We demonstrated that the agreement between the automated measurement and the manual measurement of the CSA was excellent. Such result has relevant implications, allowing for a fast, accurate, and reproducible automated measurement of CSA that would be an upgrade even for expert sonographers if implemented on the US machine. In fact, the manual measurement of the CSA is time-consuming and can increase both the intra- and inter-reader variability.

The developed algorithm, even though almost perfect in normal anatomy images analysis, has demonstrated a sub-optimal performance when asked to interpret US images with relatively infrequent anatomical variants. In particular, a bifid median nerve has been previously found in 15–18% of patients by US [36]. This represents a relevant aspect to keep in mind when interpreting the results of the present study. In fact, the impact of different anatomical variants on the performances of the algorithm may be the main obstacle to the immediate application of this software in clinical practice. Thus, future research should focus on the expansion of the dataset with US images encompassing a wider spectrum of normal anatomy at the carpal tunnel level in order to improve the algorithm performances and its generalization.

Our study has some limitations. First, the dataset is relatively small, thus not encompassing the entire spectrum of the possible anatomical variants and pathologic changes at the carpal tunnel level. Second, the US images were obtained with a high-end equipment; therefore, our results may not be generalizable if low-quality US machines/low-frequency probes are used. Moreover, the algorithm was developed and tested with US images considered of sufficient quality; thus, such a quality level represents a pre-requisite for this algorithm application. Finally, the single-center design of the study may further limit the generalizability of our results.

## Conclusions

In the present study, we developed a CNN algorithm for the localization and segmentation of the median nerve and for the automatic measurement of its CSA on US images acquired at the proximal inlet of the carpal tunnel. Such algorithm has shown excellent performances, even though future research should aim at expanding the US images dataset in order to further improve its performance in the presence of anatomical variants. This DL approach has shown very high potentiality for a fully automatic US support for CTS evaluation.

## Data Availability

The datasets used and/or analyzed during the current study are available from the corresponding author on reasonable request.

## Abbreviations

AI: Artificial intelligence
DL: Deep learning
CI: Confidence interval
CNN: Convolutional neural network
CSA: Cross-sectional area
CTS: Carpal tunnel syndrome
DSC: Dice similarity coefficient
FN: False negative
FP: False positive
FPN: Feature pyramid network
ICC: Intraclass correlation coefficient
mAP: Mean average precision
Prec: Precision
Rec: Recall
ReLU: Rectified linear unit
RPN: Region proposal network
SD: Standard deviation
TP: True positive
US: Ultrasound
